# Imprecision in the Era of Precision Medicine in Non-Small Cell Lung Cancer

**DOI:** 10.3389/fmed.2017.00039

**Published:** 2017-04-10

**Authors:** Raghav Sundar, Maxime Chénard-Poirier, Dearbhaile Catherine Collins, Timothy A. Yap

**Affiliations:** ^1^Royal Marsden Hospital, London, UK; ^2^Department of Haematology-Oncology, National University Cancer Institute of Singapore, Singapore, Singapore; ^3^The Institute of Cancer Research, London, UK

**Keywords:** precision medicine, lung cancer, targeted therapy, imprecision, clinical trials

## Abstract

Over the past decade, major advances have been made in the management of advanced non-small cell lung cancer (NSCLC). There has been a particular focus on the identification and targeting of putative driver aberrations, which has propelled NSCLC to the forefront of precision medicine. Several novel molecularly targeted agents have now achieved regulatory approval, while many others are currently in late-phase clinical trial testing. These antitumor therapies have significantly impacted the clinical outcomes of advanced NSCLC and provided patients with much hope for the future. Despite this, multiple deficiencies still exist in our knowledge of this complex disease, and further research is urgently required to overcome these critical issues. This review traces the path undertaken by the different therapeutics assessed in NSCLC and the impact of precision medicine in this disease. We also discuss the areas of “imprecision” that still exist in NSCLC and the modern hypothesis-testing studies being conducted to address these key challenges.

## Introduction

**Figure 1:**
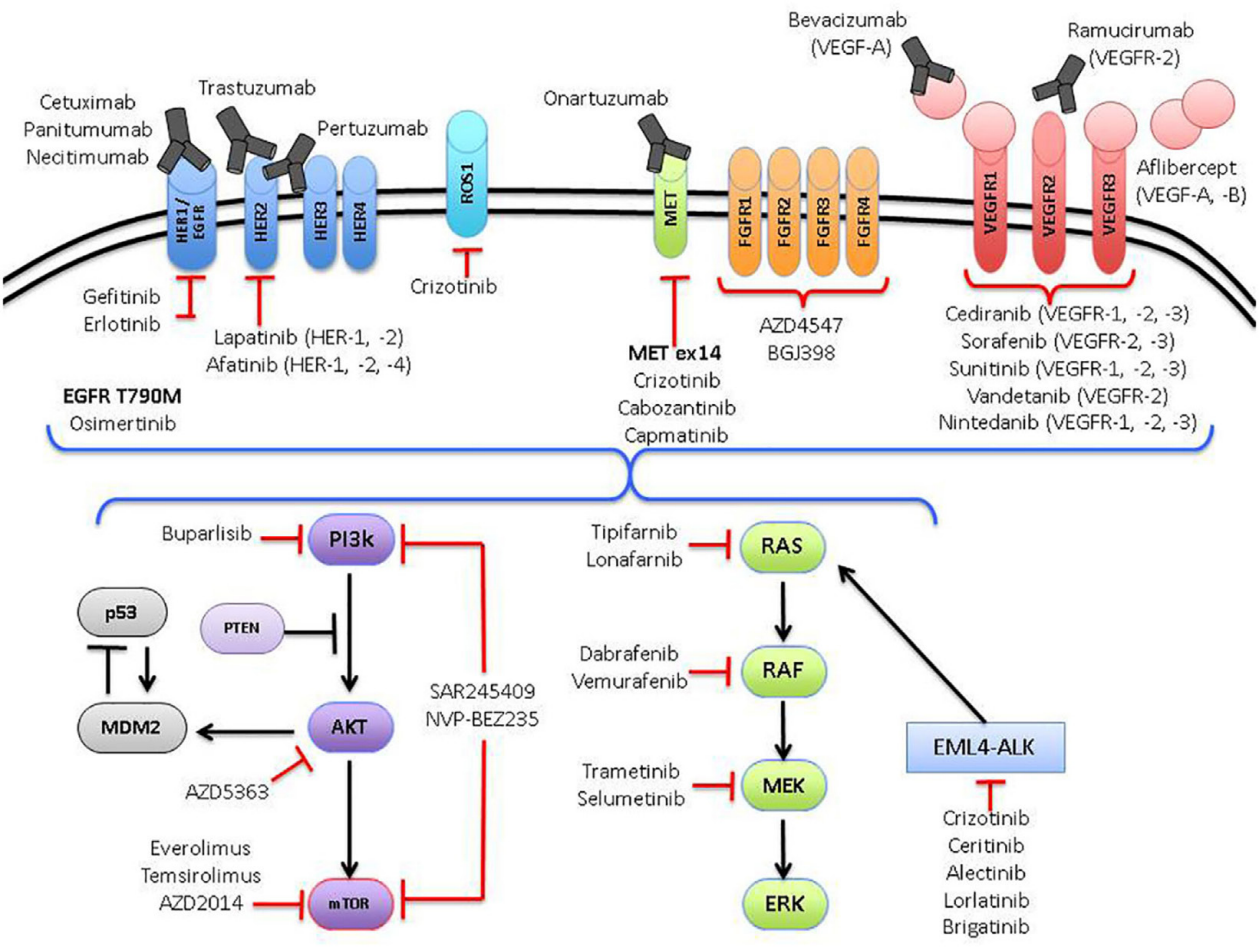
**Oncogenic pathways currently being targeted in non-small cell lung cancer**.

The management of advanced lung cancer has evolved dramatically over the past two decades. Back in the early 1990s, little was done to distinguish between the different histological subgroups of non-small cell lung cancer (NSCLC), with most trials focused on intensifying chemotherapy regimens and establishing the most effective treatments for advanced NSCLC (1), irrespective of histological subtype (2–4). Subsequently, subgroup analysis for a large randomized trial revealed critical differences in survival between patients with squamous and non-squamous histology treated with different chemotherapeutic agents (pemetrexed versus gemcitabine, in combination with cisplatin) (5). The development of tyrosine kinase inhibitors (TKIs) to epidermal growth factor receptor (*EGFR*) mutated NSCLC heralded the era of precision medicine in lung cancer. This prompted a paradigm shift toward the search for molecularly targeted agents against other putative driver aberrations in NSCLC and has led to the development of novel therapeutics matched against specific actionable aberrations, such as crizotinib (Pfizer) against *ALK* and *ROS1* aberrations (6, 7) (Figure 1).

Despite these selected successes in NSCLC and the initial promise of individualizing treatments for all patients, the management of this disease for most remains generally imprecise. Current efforts are now focused on the matching of multiple actionable drivers with targeted agents in specific disease subgroups through large basket and umbrella adaptive trials. This article describes the current state of play in the development of molecularly targeted therapies for NSCLC and addresses the successes, pitfalls, and opportunities of precision medicine in this disease.

## Food and Drug Administration (FDA)-Approved Molecularly Targeted Agents

### *EGFR* Mutations

The initial rationale for targeting EGFR in NSCLC was based on the overexpression of EGFR in NSCLC (8) and its association with worse survival (9). Initial clinical trials (IDEAL 1 and 2) involving the EGFR TKI gefitinib (AstraZeneca) were promising (10, 11) and led to accelerated FDA approval (12). However, after the failure of the drug in a large randomized phase III study (ISEL trial) (13), FDA approval for gefitinib was withdrawn. Importantly, a subgroup of patients who were non-smokers and/or of Asian descent appeared to benefit from the drug. During the same period, another EGFR TKI, erlotinib (Roche), showed a survival benefit in an unselected population of patients with refractory NSCLC (BR.21 trial) (14), which subsequently led to FDA approval. Much time and effort was spent on studying EGFR alterations, using immunohistochemistry (IHC), gene amplification, and gene-copy number, with no clear correlation with efficacy. Sequencing of receptor tyrosine kinase genes revealed somatic mutations in *EGFR* and only tumors with these mutations responded to gefitinib, while wild-type tumors did not respond (15). It took another 5 years, before the landmark IPASS trial (16) and several other pivotal phase III randomized studies (17, 18) demonstrated the importance of *EGFR* mutations as a critical driver in NSCLC, and established EGFR TKIs as the standard-of-care first-line therapy for this subgroup of patients. Gefitinib had FDA approval reinstated for first line, after a phase IV study done in the Caucasian *EGFR-*mutated population demonstrated similar responses and survival to randomized studies (19).

Afatinib (Boehringer Ingelheim), a pan-HER family small-molecule inhibitor, binds irreversibly to EGFR and is considered a second-generation EGFR TKI. Phase III trials in Western population and the Asian populations (Lux-Lung 3 and Lux-Lung 6) demonstrated a progression-free survival (PFS) benefit over platinum-based doublet therapy (20, 21). While both trials did not demonstrate an overall survival (OS) benefit for afatinib over chemotherapy, a combined analysis of both trials revealed a statistically significant OS benefit for patients with exon 19 mutations in *EGFR* (22). Afatinib has also been combined with cetuximab (Merck), a chimeric monoclonal anti-EGFR antibody and has demonstrated promising clinical activity in *EGFR-*mutant NSCLC, albeit at the cost of high rates of diarrhea and skin rash (23). Erlotinib and gefitinib have been compared head-to-head in two randomized phase III studies and revealed no significant differences in response rates and survival, suggesting equivalence between the two drugs (24, 25).

Although response to initial therapy with EGFR TKI is common, resistance to therapy is invariable and is often due to secondary mutations in *EGFR* and amplification of *MET*. The most common secondary mutation in *EGFR* is the substitution of methionine for threonine (T790M) (26). Osimertinib (Astra Zeneca), a third-generation EGFR TKI with activity against T790M was FDA approved for use in patients with NSCLC *EGFR* mutations, who have progressed on prior EGFR therapy and harbor *EGFR* T790M mutations. In a phase III study, osimertinib demonstrated a PFS benefit of 5.7 months over platinum doublet therapy (27). The role of afatinib in inhibiting *EGFR* T790M-mutant NSCLC still remains unclear in the clinic and is probably now academic given the regulatory approval of osimertinib.

### *ALK* Translocations

Inversion of the short arm of chromosome 2 leads to the joining of exons 1 to 13 of *EML4* and exons 20 to 29 of *ALK*, resulting in the EML4–ALK chimeric protein, which is known to occur in approximately 4–7% of NSCLC (28, 29). *ALK* translocations are usually mutually exclusive to *EGFR* and *KRAS* mutations (30). Crizotinib (Pfizer) was initially developed as a MET inhibitor (31), but was also found to be a potent inhibitor of ALK signal transduction (32). Compared to EGFR TKIs, ALK-inhibitor trials have been conducted primarily in biomarker-selected studies, involving patients with *ALK-*translocated NSCLC. These early to late clinical trials have demonstrated clear survival benefit and have since obtained regulatory approval for routine clinical use. Crizotinib demonstrated a PFS benefit versus chemotherapy (docetaxel or pemetrexed) in patients previously treated with a platinum doublet chemotherapy (6). Benefit in a chemotherapy-naïve population was then subsequently proven in a trial of crizotinib versus platinum/pemetrexed (33). OS benefit was not demonstrated in either trial, likely due to a cross-over effect. Despite initial antitumor responses, resistance to crizotinib invariably develops, commonly in the gatekeeper mutation L1196M, or G1269A and G1202R (34), providing the rationale for the development of second-generation ALK-inhibitors.

Ceritinib (Novartis) is 20 times more potent than crizotinib and was developed in the clinic in a small, biomarker-driven phase I study of *ALK-*translocated NSCLC, in which 66% of patients were previously treated with crizotinib, demonstrating excellent response rates of 58% and a PFS of 7 months (35), leading to FDA approval of the drug after a phase I study (a first in the modern oncology era). The efficacy of ceritinib was proven further in a phase II study (36), and phase III studies are currently ongoing (NCT01828112, NCT01828099). It should be noted that while ceritinib has shown activity against the L1196M and G1269A resistance mutations, it is ineffective against G1202R mutations (35). Brain metastases are common in NSCLC, and are often a “sanctuary site” of disease progression for patients on TKI therapy. Crizotinib has only modest cerebrospinal fluid penetration, while another second-generation ALK-inhibitor, alectinib (Genentech) has comparatively much improved activity against brain metastases (37). Two phase II studies in crizotinib-resistant *ALK-*translocated NSCLC have demonstrated significant response and disease control (38, 39). Both were single-arm studies that included *ALK*-translocated NSCLC that that failed crizotinib therapy and demonstrated response rates of 50%, leading to FDA approval for alectinib. Preliminary data were presented for a first-line Japanese study (J-ALEX) comparing alectinib with crizotinib (40) and suggested an improved PFS for alectinib over crizotinib, with better tolerance. Final data from the J-ALEX study as well as the ALEX (global) study are awaited.

Lorlatinib (Pfizer) was developed to target the G1202R-mutated population, which is resistant to crizotinib, ceritinib, and alectinib. It has demonstrated antitumor activity in patients who have progressed on two or more prior ALK-inhibitors (41). Other ALK-inhibitors that are currently in clinical trials include brigatinib (Ariad), which also targets G1202R mutations (42) and ensartinib (X-396; Xcovery), a potent second-generation inhibitor with activity against L1196M and C1156Y mutations (43). Other resistance mechanisms to ALK-inhibitors include bypass signaling through HER3 and insulin-like growth factor-1 receptor pathways, and these will probably require combination strategies to overcome such complex networks of signaling resistance (44).

### *ROS1* Translocations

ROS1 is an insulin receptor family tyrosine kinase with translocation aberrations most commonly with *CD74*, and occurs in 1–2% of patients with NSCLC (45). Aberrant ROS1 kinase activity leads to downstream signaling of the PI3K and MAPK pathways (46). As ROS1 and ALK tyrosine kinase domains have a high degree of homology, crizotinib has been shown to also inhibit ROS1 effectively (45). Clinical activity in this subgroup of patients with crizotinib included response rates of over 70% and a median duration of response of 18 months (7, 47), leading to FDA approval of crizotinib in this subgroup of patients. Ceritinib appears to show clinical activity in *ROS1*-rearranged NSCLC upon progression on crizotinib (48).

### Targeted Therapy in NSCLC Not Selected for Driver Mutations

#### Antiangiogenic Agents

High levels of vascular endothelial growth factor expression in NSCLC have been associated with a poorer prognosis, providing rationale for the use of antiangiogenic agents in this population. Several antiangiogenic agents have proven to be effective in the management of advanced NSCLC. Bevacizumab (Roche) was the first drug to show an OS benefit in combination with carboplatin and paclitaxel (E4599 trial) (49) and has been FDA approved for use in lung adenocarcinoma. However, there have been other negative phase III trials with the addition of bevacizumab to chemotherapy in the first line (50). Despite FDA approval, other drug approval bodies such as the National Institute for Clinical Excellence in the UK have not approved the use of this regimen (51). Ramucirumab (Lilly Oncology), a second-generation recombinant human monoclonal antibody targeting the vascular endothelial growth factor receptor 2 (VEGFR2), was shown to improve OS when combined with docetaxel in the second-line setting in NSCLC (52) and is FDA approved for this indication. Nintedanib (Boehringer Ingelheim), a multikinase inhibitor against VEGFR, PDGFR, and FGFR showed a survival benefit in subset analysis in patients with lung adenocarcinoma (53) and is now approved for routine use in the United Kingdom. Despite much investment spent on the development of these novel antiangiogenic agents in NSCLC, including several others currently in clinical trials, including aflibercept (Sanofi) and bavituximab (Peregerine) (54), the discovery of predictive biomarkers of response for these inhibitors remains a challenge. Several putative predictive biomarkers, including circulating VEGF-A isoform, neurophilin-1 expression, and VEGFR-1 expression, have failed to predict the antitumor effects of bevacizumab in lung cancer and other tumor types (55, 56).

#### Anti-EGFR Antibodies

There have been mixed results with clinical trials assessing anti-EGFR antibodies in advanced NSCLC. Cetuximab demonstrated an OS benefit of 11.3 months when combined with cisplatin and vinorelbine chemotherapy versus 10.1 months with cisplatin and vinorelbine (HR 0.87, *p* = 0.044) in the first-line treatment of NSCLC in the FLEX trial (57). However, there was no PFS benefit, with both arms reporting a PFS of 4.8 months. This study was followed by a negative phase III study (BMS 099 trial) (58), when cetuximab was combined with a treatment regimen of either carboplatin and paclitaxel chemotherapy or docetaxel. Similar to the FLEX study, there was no PFS benefit observed in this trial. The OS benefit between the two arms was 9.7 versus 8.4 months, which was not statistically significant. Interestingly, the magnitude of benefit between the FLEX and BMS 099 studies was similar at around 1.2 months. Based on these data, both the FDA and the European Medicines Agency rejected the use of cetuximab in the first-line setting for metastatic NSCLC in combination with platinum-based chemotherapy, based on the lack of PFS benefit and marginal improvement in OS. Apart from cetuximab, the second-generation anti-EGFR antibody necitimumab (Lilly Oncology) has been assessed in combination with cisplatin and gemcitabine in squamous NSCLC in the SQUIRE study (59). The necitimumab combination extended OS modestly from 9.9 months to 11.5 months versus cisplatin and gemcitabine chemotherapy and has since been approved by the FDA for use in first-line squamous NSCLC.

## Promising Targets in NSCLC

### MET Aberrations

*MET* amplification has gained much interest as a putative mechanism of resistance to EGFR TKI therapy. However, MET overexpression and amplification may also occur *de novo* in 50% (60) and 5% (61) of NSCLC, respectively. Tivantinib (ArQule), a small-molecule TKI, was studied in a large randomized phase III study (62) in combination with erlotinib, in patients with advanced NSCLC who had failed 1–2 lines of standard therapy. There was no improvement in OS (8.5 versus 7.8 m, *p* = 0.81), although PFS improved from 1.9 to 3.6 m (*p* < 0.01). Tivantinib was later shown to have cytotoxic activity independent of MET inhibition through microtubule disruption, similar to vincristine (63, 64). Onartuzumab (Roche), a monoclonal antibody targeting MET showed promising results in a phase II study (65), but failed to show any benefit in a large randomized phase III study when combined with erlotinib, with a median PFS of 2.7 versus 2.6 m on both arms of the study (66, 67). This study highlighted the challenges of selecting patients with truly MET-addicted NSCLC. At current time, MET overexpression based on IHC does not appear to be sufficiently robust as a predictive biomarker of response (68). In contrast, the recent impressive responses observed with crizotinib and other MET inhibitors in patients with *MET* exon 14 skipping alterations, has renewed interest in the development of MET inhibitors in NSCLC (69, 70). *MET* exon 14 aberrations occur in approximately 3–4% of non-squamous NSCLC and are hypothesized to decrease MET degradation, transforming it into an oncogenic driver (69, 71). Capmatinib (INC280, Novartis), a small-molecule inhibitor of MET, has reported responses in a case-series in patients with *MET* exon 14 skipping mutations (69). MGCD265 (Glesatinib, Mirati Therapeutics), a small-molecule inhibitor of MET and Axl, is being investigated in NSCLC with genetic alterations in MET (72). Resistance to MET inhibition can occur through secondary mutations in the *MET* kinase domain, such as *D1228N* and *Y1230C* (73, 74). High *MET* amplification also appears to be a promising predictive biomarker of response to MET inhibitors, with early studies showing antitumor responses to crizotinib in this subgroup of patients (75). In order to optimize patient benefit and accelerate the path to drug approval, future trials should include molecular profiling designed to detect MET-driven NSCLC through *MET* amplification and exon 14 skipping alterations.

### *BRAF* Mutations

These occur in about 2% of NSCLC, and like *KRAS* mutations, are more common in smokers (76). Similar to melanoma, the most common mutation is V600E in exon 15. BRAF inhibitors, such as vemurafenib (Genentech), which are approved for use in melanoma, have shown to have preliminary clinical activity in NSCLC as well (77). Of 20 patients treated, the objective response rate was 42%, median PFS was 7.3 months and 12-month OS was 66%.

### *HER2* Mutations

Compared to the more familiar *HER2-*amplification in breast and gastric cancer, *HER2* mutations occur in about 1–2% of NSCLC, most commonly in exon 20. Trastuzumab (Roche), which is standard-of-care for *HER2-*amplified breast and gastric cancers, has failed to robustly demonstrate antitumor activity in *HER2-*mutated NSCLC (78). However, afatinib, an irreversible small-molecule TKI that inhibits HER1, 2, and 4, has been shown to have clinical activity in this subgroup of patients (79). Neratinib (Puma), a pan-HER inhibitor was evaluated in combination with temsirolimus in a phase I study, with two out of six *HER2*-mutated NSCLC demonstrating a partial response (80). A phase II trial evaluating neratinib in *HER2-*mutated NSCLC is currently ongoing (NCT1827267). Dacomitinib (Pfizer), also an irreversible pan-HER TKI, demonstrated an overall response of 12% in *HER2-*mutant NSCLC in a phase II study (81).

### *RET* Translocation

*RET* translocation with genes *KIF5B, CCDC6, and NCOA4* occurs in about 1% of adenocarcinoma NSCLC (82). Cabozantinib (Exelixis) a small-molecule inhibitor of RET, MET, AXL, and VEGFR2, has shown activity in *RET*-translocated NSCLC in a phase II trial (83). Case reports have also been reported of response to vandetanib (AstraZeneca) (84).

### PI3K Pathway Aberrations

*PIK3CA* mutations have been described in 9% of squamous NSCLC (85) and are also postulated to occur as a resistance mechanism to EGFR inhibitors (86), while *AKT* mutations occur in about 5% of squamous NSCLC (87). In addition, PTEN loss occurs in approximately 20% of squamous NSCLC and 4% of lung adenocarcinoma (85). Several trials assessing mTOR, PI3K, and AKT inhibitors have been conducted to target this pathway in NSCLC. Unfortunately, most of these studies have been conducted in biomarker “unselected” populations, leading to negative results. Everolimus (Novartis), an inhibitor of mTORC1, had a response rate of 4.7%, with significant toxicities, including diarrhea (72%), rash (53%), and stomatitis (72%) (88). Another study combining everolimus with docetaxel in an unselected population had an ORR of 8%, which did not improve on the historical single agent response rates of docetaxel (89). Several novel TORC and PI3K inhibitors are currently in clinical trials, with early results already presented in abstract form. Buparlisib (Novartis) is a pan-PI3K inhibitor, which was assessed in a *PIK3CA*-activated [defined as *PIK3CA* mutation, *PTEN* mutation, or PTEN loss (less than 10% protein expression by IHC)] NSCLC population. The study reported a modest ORR of 3%, and a 12-week PFS of just 20%, leading to early termination of the study (90).

These negative findings have led to much discussion about whether such aberrations along the PI3K pathway are *bona fide* “driver” oncogenic mutations or simply “passenger” bystander mutations. However, AZD2014 (AstraZeneca), a dual TORC1 and TORC2 inhibitor, has reported early antitumor activity in patients with advanced squamous NSCLC, when combined with weekly paclitaxel, including those previously exposed to taxane chemotherapy (91). AZD5363, a potent catalytic inhibitor of all three isoforms of AKT (AKT1, 2, and 3), has demonstrated single agent activity in *AKT E17K-*mutated lung cancers, which occur in about 1% of NSCLC (92).

Targeting the PI3K pathway is more complex than inhibiting other pathways, probably because of the complex network of signaling pathways, including the disruption of negative feedback loops or development of signaling crosstalk with parallel resistance pathways.

### *FGFR1* Amplification

*FGFR1* amplifications are seen almost exclusively in smokers and occur in about 25% of squamous NSCLC (93). BGJ398 (Novartis) is a pan-FGFR inhibitor and was tested in a phase I, biomarker-selected dose-escalation study of *FGFR1*-amplified squamous NSCLC, where only 12% achieved partial responses (94). AZD4547 (AstraZeneca), a FGFR1–3 inhibitor, was assessed in a biomarker-driven group of patients with squamous NSCLC with FGFR amplification. Again, only 7% of patients had partial responses (95). Several questions have been raised on the validity of *FGFR* amplification being chosen as the predictive biomarker for these drugs and if this is indeed a true oncogenic driver (96). Importantly, high-level clonal amplification of *FGFR2* has been shown to have a differentially higher response to AZD4547 in gastric cancer (97), and this should now be assessed in lung cancer to allow for better patient selection in FGFR inhibitor trials.

### *KRAS* Mutations

*KRAS* mutations comprise approximately 25% of NSCLC, especially in smokers (98). Drugging RAS has unfortunately largely failed to date (99) and efforts to target the pathway downstream of RAS has yielded only modest results. For example, in a trial of selumetinib (AstraZeneca) in combination with docetaxel, PFS was improved from 2.1 to 5.3 months, and a trend in OS improvement (9.4 versus 5.2 m) was observed (100). In another trial of selumetinib with erlotinib, the combination of the two drugs led to increased toxicity without any improvement in ORR and PFS (101). In addition, the MEK inhibitor trametinib (Novartis) did not show any benefit in PFS or ORR when compared to docetaxel in *KRAS-*mutated lung cancer (102). Chemotherapy remains the standard-of-care for first-line metastatic *KRAS*-mutated NSCLC. A novel RAF/MEK inhibitor, RO5126766, showed promise in a phase I expansion of *KRAS*-mutated NSCLC with preliminary results being recently presented, and mature results are awaited (103).

### *DDR2* Mutations

*DDR2* mutations occur in approximately 4% of squamous cell NSCLC. DDR2 is a receptor tyrosine kinase that binds to collagen and promotes cellular proliferation. While the main target of dasatinib (Bristol-Myers Squibb) is BCR/ABL, it also inhibits DDR2 and appears to have early signals of antitumor activity in this subgroup of patients (104).

### *NTRK* Translocation

*NTRK* translocation occurs in <1% of NSCLC and include rearrangements in *NTRK1, NTRK2*, and *NTRK3*. NTRK activation leads to downstream signaling through the MAPK and PI3K pathways. Entrectinib (RXDX-101) demonstrated a durable response in a patient with NTRK1 gene rearrangement (105) and trials investigating this drug are currently on going.

## Modern Precision Medicine Trial Designs in NSCLC

**Figure 2:**
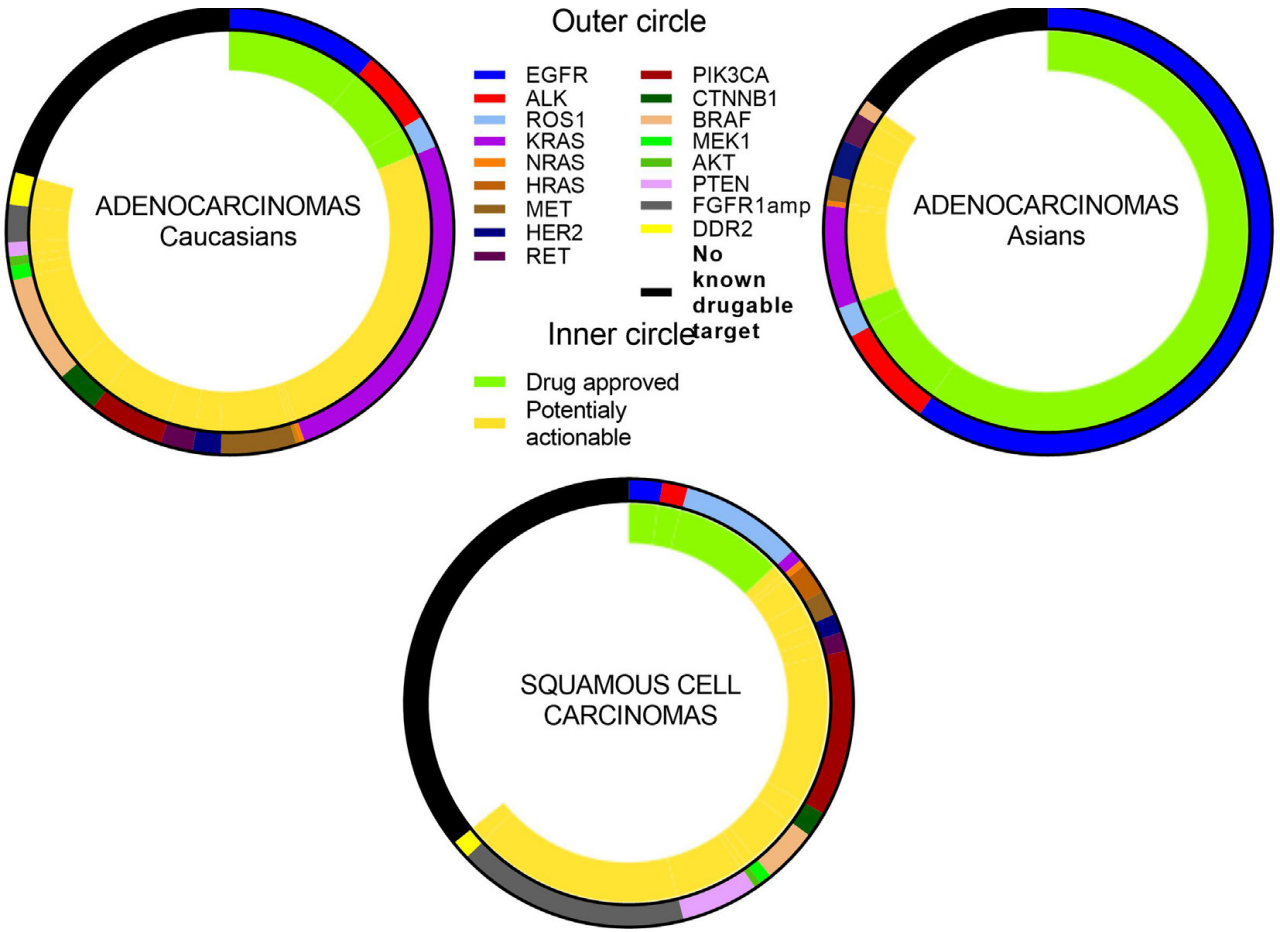
**Relative frequency of genetic abnormalities in non-small cell lung cancer lung cancer**. Note: some aberrations can occur concomitantly (not displayed in figure).

One of the main issues to address in NSCLC is that many driver aberrations only constitute a small percentage of the entire NSCLC population (Figure 2). Traditional registration trial strategies involving randomized, placebo-controlled, double-blind phase III clinical trials are, therefore, not optimal approaches and may even be considered unethical in view of the placebo control arms. Technical issues also arise as the detection of the multiple driver mutations are performed on different platforms. For example, *EGFR* mutations are usually detected by real-time polymerase chain reaction, while *ALK* rearrangements are detected by IHC and/or fluorescent *in situ* hybridization. Currently, a large number of driver aberrations in NSCLC can be assessed using large multiplexed next-generation sequencing (NGS) platforms. These are now increasingly being incorporated into clinical trials and daily clinical practice (106). Such an approach involving multiple NGS platforms abrogates issues associated with screening patients for “low frequency” genetic aberrations, especially if they are directly linked to a master protocol adaptive clinical trial. Umbrella trials assess multiple pre-specified genetic aberrations using NGS or other platforms and are matched to targeted agents, usually involving specific tumor types. Basket trials involve patients with a single or family of genetic abnormalities and are matched to targeted therapies, regardless of tumor origin (Table 1).

**Table 1:** **Modern precision medicine trials in NSCLC**.

Trial name	Precision medicine trial type	Investigational agent and target if known	Inclusion criteria	Target recruitment (*n*)	Sponsor/country	NCT identifier
BATTLE-2 Biomarker-integrated targeted therapy study	Umbrella Phase II	Group 1: erlotinib Group 2: erlotinib + MK-2206 Group 3: AZD6244 + MK-2206 Group 4: sorafenib	Stage IIIB or IV NSCLC progressed on first-line treatment	334	MD Anderson Cancer Centre, USA	NCT01248247

S1400 Lung-MAP Lung cancer master protocol	Umbrella Phase II/III	MEDI4736 (durvalumab): no active drug-biomarker option	Recurrent advanced, stage IV squamous NSCLC	10,000	SWOG/NCI, USA	NCT02154490
		AZD4547: FGFR1, FGFR2, FGFR3				
		Erlotinib ± rilotumumab: HGF/c-MET				
		Nivolumab ± ipilimumab: no active drug-biomarker option				
		Palbociclib: CDK4/6, CCND1, 2, and 3				
		Taselisib: PI3KCA expression				
		All arms are randomized to biological agent or docetaxel				

MATCH Molecular analysis for therapy choice	Basket Phase II	Afatinib: HER2; EGFR mut	Solid tumors and lymphoma post progression on standard therapy	3,000	NCI, USA	NCT02465060
		AKT inhibitor AZD5363: Akt mut				
		Binimetinib: NRAS mut in codon 12, 13, or 61				
		Crizotinib: MET amp/exon 14 del; ALK trans; ROS1 trans/inv				
		Dabrafenib (+trametinib): BRAF V600				
		Dasatinib: DDR2 S768R, I638F, or L239R mut				
		Defactinib: NF2 inactivating mut				
		FGFR inhibitor AZD4547: FGFR1–3 amp, mut, or trans				
		Nivolumab: mismatch repair deficiency				
		Osimertinib (AZD9291): EGFR T790M				
		Palbociclib: CCND1, 2, or 3 amp + Rb expression by immunohistochemistry				
		PI3Kbeta inhibitor GSK2636771: PTEN mut, del, expression, loss				
		Sunitinib maleate: cKIT exon 9,11,13, or 14 mut				
		Taselisib: PTEN loss; PI3K mut or amp without RAS mut				
		Trametinib: BRAF V600 (with dabrafenib); BRAF fusion or non-V600; NF1 mut; GNAQ or GNA11 mut				
		Trastuzumab emtansine: HER2 amp				
		Vismodegib: SMO or PTCH1 mutation				

MPACT Molecular profiling-based assignment of cancer therapy	Basket Phase II	Everolimus: PI3K pathway defect	Advanced solid tumors	700	NCI, USA	NCT01827384
		MK-1775 (Wee1 inhibitor) + carboplatin: DNA pathway repair defects				
		Temozolomide + veliparib (ABT-888; PARP inhibitor): DNA repair pathway defects				
		Trametinib DMSO: Ras/Raf/Mek pathway mut				

National lung matrix trial	Umbrella Phase II	AZD4547 (FGFR inhibitor)	Stage IIIB or IV NSCLC	620	University of Birmingham, UK	NCT02664935
		AZD2014 (MTORC1/2 inhibitor)				
		AZD5363 (AKT inhibitor)				
		AZD9291 (EGFRm + T790M + inhibitor)				
		Crizotinib (ALK/MET/ROS1 inhibitor)				
		MEDI4736 (anti-PDL1)				
		Palbociclib (CDK4/6 inhibitor)				
		Selumetinib (MEK inhibitor) + doectaxel

TAPUR Targeted agent and profiling utilization registry	Basket Phase II	Axitinib: vascular endothelial growth factor receptor (VEGFR) mut, amp, overexpression	Advanced solid tumors, multiple myeloma and B-cell non-Hodgkin lymphoma	1,030	ASCO, USA	NCT02693535
		Bosutinib: Bcr-Abl, SRC, LYN, LCK mut				
		Cetuximab: KRAS, NRAS, and BRAF wild-type				
		Crizotinib: ALK, ROS1, and MET mut				
		Dasatinib: Bcr-Abl, SRC, KIT, PDGFRB, EPHA2, FYN, LCK, YES1 mut				
		Erlotinib: EGFR mut				
		Olaparib: Germline or somatic BRCA1/BRCA2 inactivating mut; ATM mut or del				
		Palbociclib: CDKN2A/p16 loss; CDK4 and CDK6 amp				
		Pembrolizumab: POLE/POLD1 mut				
		Regorafenib: RET, VEGFR1, vascular endothelial growth factor receptor 2, VEGFR3, KIT, PDGFR-beta, RAF-1, BRAF mut/amp				
		Sunitinib: CSF1R, PDGFR, VEGFR mut				
		Temsirolimus: mTOR or TSC mut				
		Trastuzumab + pertuzumab: HER2 amp				
		Emurafenib + Cobimetinib: BRAF V600E mut				
		Vismodegib: PTCH1 del or inactivating mut				

*amp, amplification; ASCO, American Society of Clinical Oncology; del, deletion; EGFR, epidermal growth factor receptor 1; EGFRm+, EGFR-mutant; HER2, human epidermal growth factor receptor 2; inv, inversion; mut, mutation; NCI, National Cancer Institute; NSCLC, non-small cell lung cancer; SWOG, South Western Oncology Group; trans, translocation; UK, United Kingdom; USA, United States of America*.

### Umbrella Trials

#### BATTLE-2 Study

A Biomarker-Integrated Targeted Therapy Study in Previously Treated Patients with Advanced NSCLC (BATTLE-2) included patients with advanced NSCLC without sensitizing *EGFR* mutations and *ALK* fusion genes that progressed on at least one line of standard therapy (107). Two hundred patients were randomized into four arms: erlotinib, erlotinib + MK-2206 (AKT inhibitor; Merck), MK-2206 + selumetinib (MEK inhibitor), or sorafenib (Bayer), stratified for *KRAS*-mutation status. The median PFS was 2 months (95% CI, 1.9–2.8 months), median OS was 6.5 months (95% CI, 5.1–7.6 months), and 1-year survival was 28%. Only six partial responses and no complete responses were observed in this cohort of patients with a median of three prior lines of therapy. Importantly, there was no significant difference in PFS or OS between the different arms. Of note, *KRAS-*mutated patients had an improved PFS in the arms involving MK-2206 + selumetinib and sorafenib when compared with the erlotinib-containing arms.

#### Lung-MAP

A Biomarker-Driven Master Protocol for Previously Treated Squamous Cell Lung Cancer (Lung-MAP) is a study conducted in patients with squamous NSCLC, after developing disease progression on first-line platinum doublet therapy (NCT02154490) (108). Mandatory archival or fresh tumor biopsy samples must be provided for biomarker testing, which includes an NGS panel of over 200 genes (Foundation Medicine) and IHC for patient allocation to different rational therapies. Five different arms targeting PD-L1, PI3K, CDK4/6, FGFR, and c-Met pathways, involve the investigational agents durvalumab (AstraZeneca), taselisib (Genentech), palbociclib (Pfizer), AZD4547, and rilotumumab (Amgen) + erlotinib, respectively, with a standard arm of docetaxel chemotherapy. After results of rilotumumab in gastric cancer showed poor efficacy and increased toxicities, the sub-study of Lung-MAP with rilotumumab + erlotinib was withdrawn. All sub-studies included 1:1 randomization to investigational agent or docetaxel. This study is currently recruiting, and the expected accrual is 10,000 patients across the United States.

#### MATRIX Trial

The National Lung Matrix trial is non-randomized multi-arm study in the United Kingdom sponsored by University of Birmingham and Cancer Research UK (NCT02664935) (109). This study involved eight investigational arms—AZD5363 (AKT inhibitor), AZD 4547 (FGFR inhibitor), AZD2014 (mTORC1/2 inhibitor), palbocilib (CDK4/6 inhibitor; Pfizer), crizotinib, AZD9291 (third-generation EGFR inhibitor), selumetinib (MEK inhibitor) + docetaxel, and durvalumab (anti PD-L1 monoclonal antibody). Biomarker testing involves a multiplex NGS panel (Illumina) that includes various actionable mutations, which determines the allocation of patients to the appropriate investigational arms.

### Basket Trials

One of the first basket studies reported was in non-melanoma patients with *BRAF V600* mutations treated with vemurafenib (77). The results of the NSCLC cohort of this study have been described in a previous section of this article.

#### MATCH Trial

The NCI Molecular Analysis for Therapy Choice (MATCH) trial (NCT02465060) is a study that utilizes somatic genomic screening to assign patients with specific molecular aberrations to matched targeted therapy, regardless of the primary tumor site (110). This study is coordinated by the ECOG-ACRIN Cancer Research Group and involves 1,059 sites across the United States with a target recruitment of 3,000 patients. All patients must have advanced solid tumors refractory to standard therapy, undergo a mandatory fresh biopsy prior to enrolling onto the study, and to undergo a biopsy upon progression of disease. The molecular profiling assays include a targeted Ampliseq panel of 143 genes and other assays, such as IHC. The latest protocol involves 24 arms and includes agents, which have either attained FDA approval or completed trials to achieve recommended phase 2 dose (RP2D). FDA-approved drug arms include afatinib, crizotinib, osimertinib, dabrafenib (Novartis), trametinib (Novartis), ado-trastuzumab emtansine (Roche), vismodegib (Genentech), sunitinib (Pfizer), dasatinib, palbocilib, and nivolumab (Bristol-Myers Squibb). Other investigational drugs include taselisib (PI3K inhibitor), GSK2636771 (PI3K inhibitor; Glaxo-Smith Kline), defactinib (FAK inhibitor; Verastem), AZD4547 (FGFR inhibitor), AZD5363 (AKT inhibitor), and binimetinib (MEK inhibitor; Array Biopharma).

#### Molecular Profiling-Based Assignment of Cancer Therapy (MPACT) Trial

The MPACT study (NCT01827384), which is sponsored by the NCI aims to recruit 700 patients across three sites in the United States. Similar to the MATCH study, patients with advanced cancers, including NSCLC refractory to standard therapy will undergo a fresh biopsy to identify mutations in one of three pathways—MAPK, PI3K, or DNA repair. Patients with no identifiable mutations will be excluded from the study. The four treatment arms comprise veliparib (PARP inhibitor; Abbvie) + temozolomide, AZD-1775 (Wee1 inhibitor; Astra Zeneca) + carboplatin, everolimus (mTOR inhibitor), and trametinib (MEK inhibitor). The major difference between MATCH and MPACT is that patients in MPACT are randomized in a 2:1 fashion to either a “matched” arm or another arm based on their biomarker analysis. Biomarker analysis is performed on a 20-gene panel, and an informatics system, GeneMed, assists in streamlining the annotation of sequencing data, facilitating review of variant mutations, and identifying actionable mutations (110).

#### TAPUR Trial

Testing the Use of FDA-Approved Drugs That Target a Specific Abnormality in a Tumor Gene in People with Advanced Stage Cancer (TAPUR) is a trial sponsored by the American Society of Clinical Oncology with a plan to enroll 1,030 patients with advanced solid tumors refractory to standard therapy. All patients will need to harbor at least one somatic genomic variant that can be targeted by one of the drugs in the 15 arms of the study.

## Combination Treatment Strategies and Precision Medicine

Combining various anticancer agents with different mechanisms of action and minimal overlapping toxicities has been a principle applied to the management of NSCLC with varying degrees of success. In the chemotherapy era, the addition of platinum chemotherapy to other agents showed clear benefit of combination therapy (111). However, there was a limit to the number of chemotherapeutic agents that could be combined simultaneously, with triplet therapies not showing an incremental benefit over doublet regimens due to increasing toxicity (112). Combination regimen with targeted agents has innumerable permutations and is an area of active research, with many trials being conducted. A major challenge with combination targeted therapy has been synergistic toxicity, particularly involving horizontal blockade of parallel signaling pathways. These toxicities prevent dose escalation of drugs to single agent RP2Ds, leading to subtherapeutic doses and lack of target modulation due to poor pharmacokinetic exposures. While a few positive trials have emerged, many more studies have been negative (113).

### Combination Therapy with EGFR Inhibitors

During the early development of EGFR inhibitors, four large randomized phase III trials were conducted combining erlotinib and gefitinib with first-line chemotherapy in unselected patients with NSCLC. All these combination trials failed to show a survival benefit and were associated with increased toxicities (114–117). Intercalated erlotinib and chemotherapy (platinum given on day 1, gemcitabine on day 1 and 8, and erlotinib day 15–28) showed an OS benefit of 3.1 months (18.3 versus 15.2 months) in the FAST-ACT2 study in an unselected population, but subgroup analysis demonstrated that the benefit was only in the *EGFR*-mutated population (118). The combination of pemetrexed and gefitinib has demonstrated a PFS benefit of 4.9 months (15.8 versus 10.9 months) in a phase II study of *EGFR*-mutated NSCLC (119). Combining chemotherapy upon progression on EGFR TKI therapy also did not demonstrate a benefit in the phase III IMPRESS trial (120). Combination of bevacizumab with erlotinib in an *EGFR*-mutated population demonstrated a PFS benefit of 6.3 months (16 versus 9.7 months), with OS data pending (121). The rational combination of cetuximab and afatinib appear to combine with favorable response rates, albeit with higher toxicity (23). The insulin growth factor-1 receptor monoclonal antibody figitumumab (Pfizer) did not demonstrate a survival benefit and also had significantly higher toxicities when combined with erlotinib (122).

## Immune Checkpoint Inhibition and Precision Medicine

Immune checkpoint inhibition has transformed the current management landscape of NSCLC and the incorporation of this group of agents into NSCLC management is rapidly evolving. Pembrolizumab (Merck), nivolumab (Bristol-Myers Squibb), and atezolizumab (Roche) have been FDA approved for use in NSCLC. It is beyond the scope of this review to discuss immunotherapeutic strategies in detail. Cumulative data suggest PD-L1 expressing tumors benefit from both PD-1 and PD-L1 antibodies. However, several uncertainties exist, including the definition of PD-L1 positivity and variation in results observed between PD-L1 IHC assays used by different pharmaceutical companies. Currently, nivolumab is FDA approved for use in the second-line treatment of NSCLC after failure of platinum doublet therapy without biomarker selection, while pembrolizumab is FDA-approved for use in the first-line setting for tumors that express PD-L1 in at least 50% of cells. This in itself highlights the discrepancies in current clinical practice in the management of NSCLC with PD-1 inhibitors. The debate surrounding biomarker selection for immunotherapies rages on, with other novel promising predictive biomarkers of response emerging (123).

## Conclusion

The management of advanced NSCLC continues to evolve due to rapid recent advances made in precision medicine. The ultimate goal remains the identification of molecular subgroups of patients with driver aberrations who may benefit from molecularly targeted therapies that provide long-term control of NSCLC with minimal toxicities. It is now clear that there is unlikely to be a single “magic bullet” for NSCLC, and there is still a large proportion of patients with unknown or complex multiple drivers, and those harboring known driver aberrations, which are currently still not druggable. Moving forward, we will need to focus on innovative biomarker-driven trial designs with greater collaborations between academic and industry partners. There is, therefore, still much work to be done before we can truly achieve precision medicine in NSCLC.

## Author Contributions

RS: conception or design of the work; acquisition, analysis, and interpretation of data for the work; drafting the work; final approval of the version to be published; and agreement to be accountable for all aspects of the work in ensuring that questions related to the accuracy or integrity of any part of the work are appropriately investigated and resolved. MC-P: acquisition, analysis, and interpretation of data for the work; drafting the work; final approval of the version to be published; and agreement to be accountable for all aspects of the work in ensuring that questions related to the accuracy or integrity of any part of the work are appropriately investigated and resolved. DC: acquisition, analysis, and interpretation of data for the work; drafting the work; final approval of the version to be published; and agreement to be accountable for all aspects of the work in ensuring that questions related to the accuracy or integrity of any part of the work are appropriately investigated and resolved. TY: conception of the work; interpretation of data for the work; revising it critically for important intellectual content; and final approval of the version.

## Conflict of Interest Statement

TY has received research support from AstraZeneca and Merck, and has served on Advisory Boards and received travel support from Pfizer and Bristol-Myers Squibb. The authors have no other relevant affiliations or financial involvement with any organization or entity with a financial interest in or financial conflict with the subject matter or materials discussed in the manuscript apart from those disclosed.

## Abbreviations

CRUK, cancer research United Kingdom; EGFR, epidermal growth factor receptor; EMA, European medicines agency; FDA, food and drug administration; FISH, fluorescent *in situ* hybridization; IHC, immunohistochemistry; NGS, next generation sequencing; NICE, national institute for clinical excellence; NSCLC, non-small cell lung cancer; OS, overall survival; PCR, polymerase chain reaction; PFS, progression-free survival; RP2D, recommended phase 2 dose; TKI, tyrosine kinase inhibitor; VEGFR, vascular endothelial growth factor receptor.
